# Biocontrol potential of *Pseudomonas rhodesiae* GC-7 against the root-knot nematode *Meloidogyne graminicola* through both antagonistic effects and induced plant resistance

**DOI:** 10.3389/fmicb.2022.1025727

**Published:** 2022-10-13

**Authors:** Shan Ye, Rui Yan, Xinwen Li, Yufeng Lin, Zhuhong Yang, Yihang Ma, Zhong Ding

**Affiliations:** ^1^College of Plant Protection, Hunan Agricultural University, Changsha, Hunan, China; ^2^Hunan Provincial Engineering & Technology Research Center for Biopesticide and Formulation Processing, Changsha, Hunan, China; ^3^Agriculture and Rural Department of Hunan Province, Plant Protection and Inspection Station, Changsha, Hunan, China; ^4^Department of Chemical Metrology and Reference Materials, Hunan Institute of Metrology and Test, Changsha, Hunan, China

**Keywords:** *Meloidogyne graminicola*, biological control, nematicidal activity, defense enzyme, systemic resistance

## Abstract

Plant-parasitic nematodes (PPNs) cause serious damage to agricultural production worldwide. Currently, because of a lack of effective and environmental-friendly chemical nematicides, the use of microbial nematicides has been proposed as an eco-friendly management strategy to control PPNs. A nematicidal bacterium GC-7 was originally isolated from the rice rhizosphere, and was identified as *Pseudomonas rhodesiae.* Treatment with the fermentation supernatant of GC-7 *in vitro* showed a highly lethal effect on second-stage juveniles of *Meloidogyne graminicola*, with the mortality rate increasing to 95.82% at 24 h and egg hatching significantly inhibited, with a hatch inhibition rate of 60.65% at 96 h. The bacterium significantly reduced the level of damage caused by *M. graminicola* infestations to rice (*Oryza sativa*) in greenhouse and field experiments. Under greenhouse conditions, the GC-7 culture efficiently reduced the gall index and nematode population in rice roots and soils, as well as inhibited nematode development compared to the control. Under field conditions, application of the GC-7 consistently showed a high biocontrol efficacy against *M. graminicola* (with a control efficiency of 58.85%) and promoted plant growth. In addition, the inoculation of GC-7 in *M. graminicola*-infested rice plant fields significantly suppressed final nematode populations in soil under natural conditions. Furthermore, activities of plant defense-related enzymes, peroxidase, polyphenol oxidase, and phenylalanine ammonia-lyase were remarkably increased in plant roots treated with GC-7 compared with roots that were challenge to *M. graminicola*. Moreover, quantitative real-time PCR analysis showed that GC-7 significantly enhanced the expression of defense genes (*PR1a*, *WRKY45*, *JaMYB*, *AOS2*, *ERF1*, and *ACS1*) related to salicylic acid, jasmonic acid, and ethylene signaling pathways in rice roots after inoculation with GC-7 at different levels. The results indicated that GC-7 could be an effective biological component in the integrated management of *M. graminicola* infecting rice.

## Introduction

Plant-parasitic nematodes (PPNs) are one of the most destructive groups of soilborne pathogens and are responsible for annual agricultural losses estimated at USD 358.24 billion worldwide in past years (Abad et al., 2008; Abd-Elgawad, 2022). The most damaging and yield-limiting group among PPNs are the root-knot nematodes (RKNs), which have a broad host range, including many economically important crops (Abad et al., 2003; Forghani and Hajihassani, 2020). The rice RKN *Meloidogyne graminicola* is one of the most devastating pests of rice, causing substantial yield losses in rice-producing areas, especially in Asia (De Waele and Elsen, 2007; Kyndt et al., 2012; Mantelin et al., 2017). *M. graminicola* is an obligate, sedentary endoparasite that occurs across a range of different rice ecosystems. Although it cannot penetrate rice roots in flooded soils, this RKN can survive long periods in anoxic environments and rapidly reinvade roots whenever soils are drained (Bridge and Page, 1982). The infective second-stage juvenile (J2) of *M. graminicola* penetrates rice roots and induces the formation of giant cells as nutrition resource throughout its life cycle. The infection is characterized by hook-shaped galls (root-knots) mainly on the root tips (Kyndt et al., 2013). Once established in the roots, J2s become sedentary and undergo three molts to become third (J3) and fourth stages (J4) and adult stage. Females remain in the galled roots, and laying eggs inside the root. On average, *M. graminicola* complete life cycle in about 19 to 27 days during the early summer, but the period can extend by 5 to 12 days (Khan et al., 2021; Rusinque et al., 2021). *M. graminicola* is difficult to control because it has a short generation and high reproduction rate (Jang et al., 2016). At present, the main strategy for controlling PPNs relies on chemical nematicides; however, they are often highly toxic to human health and the environment, causing multi-drug resistance in nematodes (Medina-Canales et al., 2019; Rajasekharan et al., 2020). Therefore, environmentally-friendly treatments targeted towards nematodes are urgently needed.

Microorganisms have shown great potential as biological agents for controlling nematode infections. Bacteria, in particular, have received considerable attention (Abd-Elgawad, 2021; Khanna et al., 2021; Migunova and Sasanelli, 2021; Zhao et al., 2021). In recent years, studies have shown the efficacy of several bacteria to control nematodes, and the use of plant growth-promoting rhizobacteria (PGPR) is considered as the most applicable and promising strategy for PPN biocontrol (Hu et al., 2017; Zhao et al., 2018; Liu et al., 2020). Previous studies have shown that *Bacillus cereus* and *Burkholderia arboris* isolated from the plants’ rhizosphere significantly induced mortality in J2s of *Meloidogyne incognita*, and markedly reduced nematode infection in host plant roots (Yin et al., 2021; Zhang et al., 2022). Similarly, *Bacillus altitudinis* can promote plant growth and showed high nematicidal activity against *Meloidogyne javanica* (Antil et al., 2022). Moreover, *Bacillus megaterium* and *Klebsiella pneumoniae* significantly inhibit the invasion, development, and reproduction of *Heterodera glycines* by inducing systemic resistance, promoting soybean growth (Liu et al., 2018; Zhou et al., 2021). So far, there are few microbial biological based nematicides, *Bacillus subtilis* and *B. amyloliquefacien*s are prevalent in the market of products used to promote plant growth and biological control (Migunova and Sasanelli, 2021). However, there was some limitations, including their relative low efficiency and high inconsistency in agricultural environments (Liang et al., 2019).

Biocontrol bacteria inhibit nematode infection *via* various mechanisms, including direct antagonisms (e.g., predation, competition for nutrients, and release of toxic metabolites) and indirect antagonisms through the induction of host systemic resistance (Bukhat et al., 2020; Forghani and Hajihassani, 2020). Typically, plants have two types of induced resistance, termed induced systemic resistance (ISR) and systemic acquired resistance (SAR) (Vallad and Goodman, 2004). ISR is activated by nonpathogenic rhizobacteria such as specific PGPR and relies on the jasmonic acid (JA)/ethylene (ET) signaling pathways in plants (Subedi et al., 2020). Generally, SAR is caused by different necrotizing pathogens and is dependent on the signaling molecule salicylic acid (SA; Conn et al., 2008). A previous study indicated that *B. cereus* Bc-cm103 activated the defense-responsive genes related to the SA, JA, and ET signaling pathways in host plants in response to *M. incognita* (Yin et al., 2021). Ghahremani et al. (2020) reported that *Bacillus firmus* I-1582 can degrade *M. incognita* eggs by inducing systemic resistance in tomato. The expression of JA and SA pathway-related genes were upregulated at different times in plants inoculated with I-1582 (Ghahremani et al., 2020). In addition, the development of inducible resistance in host plants is associated with enhanced activities of plant enzymes, including phenylalanine ammonia lyase (PAL), polyphenol oxidase (PPO), and peroxidase (POD) (Amil-Ruiz et al., 2011). Several studies show the ability of beneficial microbes to stimulate the activity of defense enzymes in host plants in response to pathogen infection (Narendra Babu et al., 2015; Salla et al., 2016; de Oliveira et al., 2021; Yan et al., 2021). The PGPR *Bacillus* spp. suppressed *Pyricularia oryzae* infection in rice by elevating the activity of antioxidant enzymes (PAL, PPO, and POD) (Rais et al., 2017). Similarly, *Pseudomonas* spp. effectively improved the growth parameters and inhibited the nematode infection by upregulating the activities of defense enzymes (SOD, POD, and PPO) (Sharma and Sharma, 2016; Khanna et al., 2019). *Streptomyces* spp. have also been reported to markedly increase PPO activity in RKN-exposed tomato plants (Ma et al., 2017).

Despite *M. graminicola*’s widespread occurrence in Asian rice production systems, the research carried out on its biological control remains limited. To date, only a few fungi and bacteria have been reported to possess antagonistic potential against *M. graminicola* and reduce the number of root knots (Padgham and Sikora, 2007; Le et al., 2016; Haque et al., 2018; Liu et al., 2019; Yang et al., 2020; Khan et al., 2021). Identifying novel nematicidal microorganisms and investigating their underlying mechanism are necessary for the biological control of *M. graminicola* and are of vital practical and economic significance. The *Pseudomonas* is one of the most frequently studied groups of bacteria used for biological control agents. The genus *Pseudomonas* is gram-negative, rod-shaped straight or slightly curved cells with polar flagella (Sah et al., 2021). Researchers have indicated that biological control by *Pseudomonas* spp. involves a variety of mechanisms, including antibiosis, nutrient competition, and induction of host systemic resistance (Walsh et al., 2001). *Pseudomonas rhodesiae* is a strain that promotes plant growth and biocontrol of tomato and rice diseases (Romero et al., 2016; Forghani and Hajihassani, 2020). However, reports of *P. rhodesiae* antagonistic against *M. graminicola* are still lacking.

The objectives of the present study were to screen for a novel effective bacterial isolate against *M. graminicola,* and investigate the effects of *P. rhodesiae* GC-7 on *M. graminicola* eggs and J2s through *in vitro* experiments. The study also evaluated the biocontrol potential of GC-7 under greenhouse and field conditions and assessed the effect of GC-7 inoculation on plant growth and on nematode infestation and development. In addition, the expression levels of defense genes related to the SA, JA, and ET pathways and the enzymatic activities of PAL, POD, and PPO in the roots of rice induced by isolate GC-7 were examined to explore the mechanism by which GC-7 suppressed *M. graminicola*.

## Materials and methods

### Isolation and identification of isolate GC-7

Isolate GC-7 was isolated from the rhizosphere soil of a rice field in Changsha city, Hunan Province, China using the serial dilution technique (up to 10^−7^-fold). A single bacterial colony was selected, streaked on beef extract-peptone medium (NA) (1% tryptone, 0.3% beef extract, 0.5% NaCl, 1.5% agar), and incubated for 24 h at 30°C to obtain the pure isolate. The bacteria were then cultured in beef extract-peptone broth (NB) at 30°C, with shaking at 200 rpm for 48 h (approximately 2 × 10^9^ cfu/ml) for further experiments.

Routine physiological and biochemical tests of isolate GC-7 were performed according to Bergey’s Manual of Determinative Bacteriology (8^th^ Ed.). They included gram staining, the methyl red (MR) Voges-Proskauer (VP), and indole tests, testing for oxidase and catalase activity, nitrate reduction, citrate utilization, starch and gelatin hydrolysis, as well as utilization of carbon sources. The identity of GC-7 was further confirmed through 16S rRNA and *gyrB* gene sequencing. Bacterial genomic DNA was extracted using the Bacteria Genomic DNA Extraction Kit (Takara, Dalian, China) according to the manufacturer’s instructions. The 16S rRNA gene fragment of this isolate was amplified *via* PCR using the universal bacterial primers 27F (AGAGTTTGAT CCTGGCTCAG) and 1492R (GGTTACCTTG TTACGACTT). The *gyrB* gene was amplified by using the primers UP-f 5′-AGCAGGGTACGGATGTGCGAGCCRTCNACRTCN GCRTCNGTCAT-3′ and UP-r 5′-GAAGTCATCATGACCGTTCTGCAYGCNGGNGGNAARTTYGA-3′ (Yamamoto and Harayama, 1995). The PCR products were sequenced by Shenggong Biotechnology Co. Ltd., Shanghai, China. The GC-7 sequence was analyzed *via* a BLASTN search in the National Center for Biotechnology Information (NCBI)^1^ database. The partial 16S rRNA and *gyrB* sequences were submitted to GenBank (NCBI) to obtain the accession number. The alignment of sequences and construction of the phylogenetic tree were performed using Clustal X 2.1 (Thompson et al., 1997) and MEGA 7.0 (Kumar et al., 2016), respectively.

### Cultivation of rice plants and preparation of nematode inoculum

Rice (*Oryza sativa* variety Nipponbare) seeds were obtained from the United States Department of Agriculture (GSOR-100). Seeds were germinated on wet filter paper for 4 days at 30°C, and seedlings were transferred to synthetic absorbent polymer SAP-substrate and further grown in a greenhouse at 28 ± 2°C under a 14-h photoperiod and 70–75% relative humidity (Reversat et al., 1999). Plants were watered with distilled water twice per week and fertilized once per week with 10 ml of Hoagland solution. A population of *M. graminicola* isolated from lowland rice in Pingjiang County, Hunan Province, China was maintained on susceptible *O. sativa* variety Nipponbare in a greenhouse in ChangSha. Infected roots and root galls were cut into pieces, and nematode eggs were isolated from the root galls. The egg masses were surface-sterilized with 1% sodium hypochlorite for 1 min and placed on double-layered tissue paper in a Baermann funnel containing distilled water for 3–5 days at 25°C to obtain second-stage juveniles (J2s). The freshly hatched J2s were used for *in vitro* assays and pot-based experiments.

### Nematicidal activity of *Pseudomonas rhodesiae* GC-7 *in vitro*

*Pseudomonas rhodesiae* isolate GC-7 was cultured in NB at 30°C on a shaker at 200 rpm for 48 h. The bacterial fermentation broth (2 × 10^9^ cfu/ml) was centrifuged at 10,000 rpm for 10 min, and the crude supernatant was passed through a 0.22-μm nitrocellulose filter to obtain the cell-free supernatant. The GC-7 fermentation supernatant was diluted with sterile water to 10, 20, and 50% (v/v) concentrations. The NB medium was diluted to a concentration of 10, 20, and 50% with sterile water.

#### Lethal activity assay of isolate GC-7 against J2s of *Meloidogyne graminicola*

The lethal activity of isolate GC-7 to J2s of *M. graminicola* was determined. The assay of mortality was performed in a 24-well plate. Each well contained approximately 100 J2s of *M. graminicola* and 1 ml of the different concentrations of GC-7 supernatant, separately. Treatments with different concentrations of NB medium were included as the control. The plates were incubated at 28°C. After 24 h, the dead and living nematodes were counted using an inverted microscope (Olympus, Tokyo, Japan). A nematode that was malformed, immobile, or motionless even when probed with a fine needle was deemed dead (Cayrol et al., 1989). Each treatment had six replicates and was performed three times independently. Mortality was calculated according to the following formula:


$$\begin{array}{l} J2s\;\mathrm{mortality}\;\left(\%\right)\\ =\left(\mathrm{The}\;\mathrm{number}\;\mathrm{of}\;\mathrm{dead}\;J2s/\mathrm{The}\;\mathrm{Number}\;\mathrm{of}\;\mathrm{total}\;J2s\right)\times 100 \end{array}$$

#### Inhibition of egg hatching by isolate GC-7 *in vitro*

The effect of isolate GC-7 on egg hatching was tested in 24-well plates, using a method similar to the J2 mortality test described previously. One hundred sterilized eggs were mixed with 1 ml of the 50% GC-7 fermentation supernatant. Eggs mixed in 1 ml of 50% NB medium were used as control. The plates were covered and incubated at 28°C. The number of unhatched eggs was counted using a microscope at 24 h, 48 h, 72 h, and 96 h after incubation. The experiment had six replicates and the test was repeated three times. The egg hatching rate and hatch inhibition rate were calculated using the following formulae:


$$\begin{array}{l} \mathrm{Egg}\;\mathrm{hatching}\;\mathrm{rate}\;\left(\%\right)\\ =\left(\mathrm{Number}\;\mathrm{of}\;\mathrm{hatched}\;\mathrm{eggs}/\mathrm{Total}\;\mathrm{number}\;\mathrm{of}\;\mathrm{eggs}\right)\times 100 \end{array}$$

$$\begin{array}{l} \begin{array}{l} \mathrm{Hatching}\;\mathrm{inhibition}\;\mathrm{rate}\;\left(\%\right)\\ =(\mathrm{The}\;\mathrm{number}\;\mathrm{of}\;\mathrm{hatched eggs in the control}\\ \;-\mathrm{The}\;\mathrm{number}\;\mathrm{of}\;\mathrm{hatched}\;\mathrm{eggs}\; \end{array}\\ \begin{array}{l} \;\mathrm{in}\;\mathrm{the}\;\mathrm{bacteria}-\mathrm{treated group})\\ \;/\mathrm{The number of hatched eggs in the control}\times 100 \end{array} \end{array}$$

### Effect of isolate GC-7 on infection by *Meloidogyne graminicola* J2s

*Meloidogyne graminicola* were inocubated on rice seedlings (*Oryza sativa* variety Nipponbare) treated with GC-7 to determine the direct effect of isolate GC-7 on the infectivity of nematodes. The roots of rice seedlings were immersed in a 50% GC-7 isolate fermentation culture (1 × 10^9^ cfu/ml) for 8 h. Roots soaked in NB medium for 8 h were used as controls. Pluronic F-127 powder (Sigma Aldrich, St Louis, United States) was added to sterile water and allowed to dissolve with stirring at 4°C for 24 h. Next, treated rice seedlings were placed in F127 gel plot, then 100 μl of *M. graminicola* suspension (1,000 vigorous J2s/mL) was added surrounding to each seedling. The F127 plot was incubated in greenhouse previously described. At 5 and 14 days post inoculation (dpi), the plant roots were carefully collected and stained using the NaOCl-acid fuchsin method (Bridge and Page, 1982). The total number of nematodes inside root galls per rice seedling was counted using a stereomicroscope (Nikon, Tokyo, Japan). The number of nematodes at different life stages was determined using a stereomicroscope to calculate their ratio and analyze the effect of GC-7 on nematode development. Each treatment had four replicates and was performed three times independently.

### Biocontrol efficacy of isolate GC-7 against *Meloidogyne graminicola* and the biomass changes of rice plants in the greenhouse

A pot-based validation experiment was conducted under greenhouse conditions at Hunan Agricultural University, China. Bacteria was prepared as described above. The bacterial fermentation broth was cultured to approximately 2 × 10^9^ cfu/ml, then diluted to obtain concentrations of 50% (1 × 10^9^ cfu/ml), 20% (4 × 10^8^ cfu/ml), and 10% (2 × 10^8^ cfu/ml). Seeds were treated as described previously. Five two-leaf stage rice seedings were transplanted into sterile soil in each pot (15 cm in diameter and 10 cm in height), it is about 1 kg of soil per pot. Different concentrations of the bacterial fermentation broth (40 ml each) and fluopyram (0.33 g a.i./L, Lufta, Bayer Crop Science, China) were added to each pot. An equal amount of sterile water was added to the pots as a control. Two days after inoculation with the bacterial fermentation broth, 2 ml of *M. graminicola* suspension (750 vigorous J2s/mL) was drenched into each pot. All pots were arranged in a completely randomized block design on a bench in the greenhouse, and each treatment consisted of four replicates. The rice plants were uprooted and washed free of adhering soil 21 days post inoculation. The number of root galls on the rice plants were examined to assess nematode penetration. The disease index of the roots was confirmed using the 0–10 scale described by Bridge and Page (Bridge and Page, 1980). Gall indices and biocontrol efficacy were calculated as follows:


$$\begin{array}{l} \begin{array}{l} \mathrm{Gall index}\;\left(\%\right)\\ =[\sum \left(\mathrm{The number of diseased plants in each grade}\right)\\ \;/(\mathrm{Total number of plants investigated}\; \end{array}\\ \;\times \mathrm{The highest grade})]\times 100 \end{array}$$

$$\begin{array}{l} \begin{array}{l} Bio-\mathrm{control efficiency}\;\left(\%\right)\\ =\left(\mathrm{Gall index in the control}-\mathrm{Gall index in the treated group}\right) \end{array}\\ \;/\left(\mathrm{Gall index in the control}\right)\times 100 \end{array}$$

Soil samples from each pot were examined to obtain the actual frequency and relative number of nematodes and eggs in the soil. The soil was mixed, and the sucrose solution-elutriation-centrifugation method was used to extract soil nematodes and eggs from approximately 100 g of fresh soil (Li et al., 2016). The total number of nematodes and eggs in each soil sample was counted under a dissecting microscope, and a statistical analysis was performed. Biomass values, including total length, root length, and fresh weight of the entire plants and roots, were measured to survey the promotion of plant growth.

### Biocontrol efficiency of isolate GC-7 against *Meloidogyne graminicola* in the field

Field trials were carried out in a field infested with *M. graminicola* in Pingjiang County City, Hunan Province, China (113.67 E, 28.57 N) in 2021. The soil in the field was a sandy loam containing 55.6% sand, 9.2% silt and 36.4% clay, with a pH of 5.0, a water holding capacity of 30%, and an organic matter content of 1.0%. The initial *M. graminicola* population consisted of 393 ± 32 J2/100g soil. The experiment consisted of three treatments: fermentation broth GC-7 (2 × 10^9^ cfu/ml), 41.7% fluopyram SC (250.2 g a.i/ha; Lufta, Bayer Crop Science, China), and water (as control). Sprouted seeds of the rice variety HuangHuaZhan were sown in the fields and irrigated with different treatments (750 l/ha). The experiment was set up in a completely randomized block design, and each treatment was applied to four replicated plots 5.0 m long and 3.0 m wide. The trial was fertilized with 375 kg/ha carbamide and 750 kg/ha diammonium phosphate 2 days before sowing, and with 75 kg/ha carbamide and 75 kg/ha potassium chloride at the middle tillering stage. After 60 days, 15 seedlings were randomly selected (using the Z-shaped sampling method) from each plot, a total of 60 seedlings were obtained from each treatment.

The rice plants were observed and disease index of the roots was recorded as described above. The plants were also used to record the effect of the GC-7 isolate on the following growth parameters: shoot and root length (cm), shoot and root fresh weight (g), and total chlorophyll content (Konica Minolta, Inc., SPAD-502 Plus, Japan). At the time of harvest (4 months after planting), the grain yield was recorded and the rhizosphere soil samples randomly selected from each plot were returned to the laboratory. The nematodes and eggs in each 100 g soil sample were isolated and counted as described above.

### Resistance-related enzymes assays

Rice seedlings were treated with different concentrations of isolate GC-7 and grown as described for the pot-based experiment to detect resistance-related enzymes. The experiment consisted of five treatments: rice seedlings treated with (1) sterile water (CK), (2) sterile water and inoculated with *M. graminicola* J2 (J2), (3) 50% GC-7 and inoculated with *M. graminicola* (50% GC-7 + J2), (4) 20% GC-7 and inoculated with *M. graminicola* (20% GC-7 + J2), and (5) 10% GC-7 and inoculated with *M. graminicola* (10% GC-7 + J2).

Defense-related enzymes, including PAL, PPO, and POD, were quantified from rice plants grown under pot conditions after 2 days, 4 days, 6 days, and 8 days of nematode inoculation. Plants were carefully uprooted, causing no damage to root tissues. The activities of PAL, PPO, and POD were extracted and measured from fresh rice root samples using commercial assay kits (Nanjing Jiancheng Bioengineering Institute, China).

PPO activity was measured using the assay kit based on the principle that PPO can catalyze quinone production from the substrate phenol, which is characterized by light absorption at 420 nm. PPO activity was calculated by the decrease in absorbance at 420 nm; one unit (U) of PPO activity was defined as a change in absorbance by 0.01 for 1 g fresh weight (FW) per minute in the 1-mL reaction system.

PAL catalyzes the decomposition of L-phenylalanine into trans-cinnamic acid and ammonia. The maximum absorption value for trans-cinnamic acid was obtained at 290 nm, and PAL activity was calculated by measuring the change in absorbance value at 290 nm. One unit of PAL activity was defined as a change in absorbance by 0.1 for 1 g FW per minute in the 1-mL reaction system. The obtained values for absorbance were converted into enzyme activity according to the formula used for assay kits.

POD activity was measured based on the change of absorbance at 420 nm by catalyzing H_2_O_2_. One unit was defined as the amount of enzyme which was catalyzed and generated 1 μg substrate by 1 g fresh weight tissues in the reaction system at 37°C. POD activity was calculated as the formula according to POD assay kit. The enzymes were expressed as U/g FW, and the experiment was performed in three replicates.

### RNA extraction and gene expression analysis

Rice seedlings were treated with isolate GC-7 and grown as previously described, with sterile water used as control. The seedlings were carefully drenched with 40 ml of *P. rhodesiae* GC-7 (1 × 10^9^ cfu/ml). Each individual pot was inoculated with approximately 1,500 freshly hatched *M. graminicola* J2s 2 days later and it is about 1 kg of soil per pot. The experiment consisted of four treatments: rice seedlings treated with (1) sterile water as the non-treated control (CK), (2) sterile water and inoculated with J2 (J2), (3) GC-7 (GC-7), and (4) GC-7 and inoculated with *M. graminicola* (GC-7 + J2). To measure the transcript levels of defense-related genes in real time, whole rice roots were collected at 1 d and 4 d after nematode inoculation, frozen immediately in liquid nitrogen, and stored at −80°C until further use. Total RNA was extracted from the rice roots using the MiniBEST Universal RNA Extraction kit (TaKaRa, Dalian, China). Total RNA from each sample (1 μg) was reverse-transcribed using the PrimeScript RT Reagent kit (TaKaRa, Dalian, China). Quantitative real-time RT-PCR assays were performed using gene-specific primers for the genes Pathogenesis-related 1a (*PR1a*), WRKY TF 45 (*WRKY45*), Allene oxide synthase2 (*AOS2),* JA-inducible Myb TF (*JaMYB*), Aminocyclopropane-1-carboxylic acid synthase 1 (*ACS1*) and Ethylene-responsive factor 1 (*ERF1*) (Takaki et al., 2015; Leonetti et al., 2017; Salman et al., 2022), described in Supplementary Table S1. Quantitative real-time PCR (qRT-PCR) was performed in a CFX Connect Real-Time PCR Detection System (Bio-Rad, California, United States) using a SYBR green I quantitative PCR master mix (TaKaRa, Dalian, China). The reaction conditions were 95°C for 30 s followed by 40 cycles at 95°C for 5 s, and 60°C for 30 s. A melting curve analysis was performed after 40 cycles to confirm that a single product was present for each reaction. qRT-PCR was performed on three biological replicates and each reaction was replicated three times. Expression of the rice gene actin was used as an internal reference gene, and the data were quantified using the 2^−ΔΔct^ method (Livak and Schmittgen, 2001).

### Statistical analysis

Statistical analysis was performed with SPSS Statistics version 20.0.0 (International Business Machines Corporation, United States). Both data normality and homogeneity of variances were assessed. Ninety-five percent fiducial limits of the experiment data were determined *via* probit analysis. For multiple-group comparison, ANOVA was performed followed by Tukey’s multiple comparison test. Statistical differences were considered significant at *p* ≤ 0.05. Different lowercase letters indicate significant differences between treatments (*p* < 0.05).

## Results

### Isolation and characterization of bacterium *Pseudomonas rhodesiae* isolate GC-7

GC-7 colonies grown on nutrient agar were round, white, and non-transparent, with a smooth surface. The bacterium had Gram-negative staining reaction with a rod-shaped structure. The detailed morphological and physiological characteristics are summarized in Table 1.

**Table 1:** Morphological and physiological characteristics of isolate GC-7.

Characteristics	Reaction	Characteristics	Reaction
Colony color	White	Citrate utilization	+
shape	Rod shaped	Indole experiment	−
Gram-stain	−	Hydrolysis of starch	+
Oxidase activity	+	Decomposition of casein	+
Catalase activity	+	Gelatin liquefaction	+
Nitrate reduction	+	Carbohydrate utilization	
Methyl red test	+	D-Glucose	+
Voges-Proskauer test	−	Lactose	−
Anaerobic growth	−	Sucrose	+

+, positive reaction; −, negative reaction.

**Figure 1:**
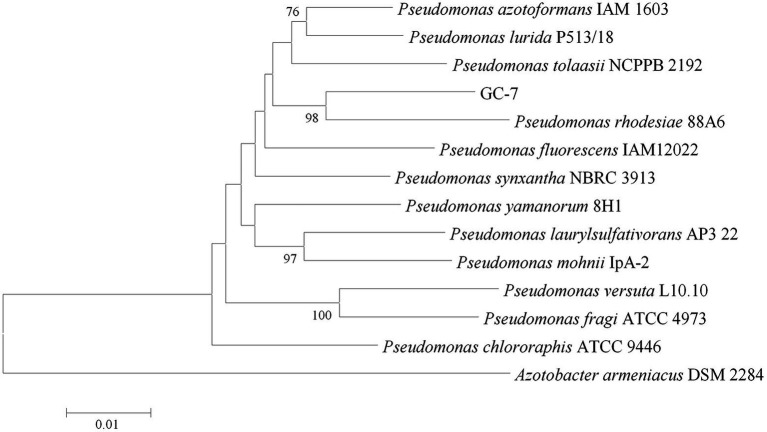
Phylogenetic tree of isolate GC-7 based on 16S rRNA and *gyrB* gene sequences. The neighbor-joining method was used to construct the tree, which shows the relationships between isolate GC-7 and closely related species. Only values greater than 50% are provided. The scale bar indicates 0.01 nucleotide substitutions per nucleotide position. The numbers at the nodes represent percentages after 1,000 bootstrap replicates.

Furthermore, identification was confirmed using 16S rRNA and *gryB* analysis. The nucleotide sequences of 16S rRNA (accession number OP160002.1) and *gryB* (accession number OP429596) deposited in GenBank of NCBI. According to a phylogenetic tree constructed using the neighbor-joining method, isolate GC-7 was found most closely related to *P. rhodesiae* (Figure 1). Based on the phylogenetic trees and morphological features, isolate GC-7 was identified as *P. rhodesiae* and was deposited at the China Center for Type Culture Collection (CCTCC) under accession number CCTCC M 2020346.

### Evaluation of the nematicidal activity of isolate GC-7 *in vitro*

**Figure 2:**
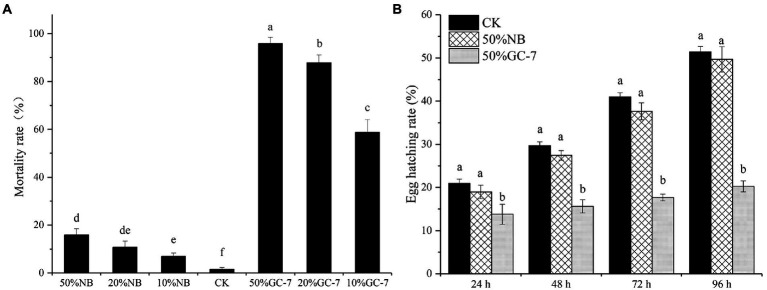
Evaluation of the nematicidal activity of *Pseudomonas rhodesiae* GC-7 *in vitro*. **(A)** Effects of isolate GC-7 on second stage juveniles (J2s) mortality of *M. graminicola in vitro*. Mortality rates were assessed by *M. graminicola* J2 incubated in fermentation cell-free supernatant of GC-7 at different concentrations in 24-well plates at 28°C for 24 h. J2 suspension mixed with different concentrations of NB medium, and sterilized water was used as control (CK). **(B)** The egg hatching rate of *M. graminicola* exposed to *Pseudomonas* sp. GC-7 cell-free supernatant *in vitro*. The egg hatching rate was evaluated by treating approximately 100 eggs with 50% GC-7 supernatant for 24 h, 48 h, 72 h, and 96 h at 28°C. The negative control was sterilized water. The results are from three independent experiments, each containing four replicates. The data were analyzed by Tukey’s multiple range test (*p <* 0.05) using SPSS software. Error bars represent standard deviation, different letters represent a significant difference at *p <* 0.05.

To determine whether *P. rhodesiae* GC-7 has nematicidal activity against *M. graminicola*, a mortality assay was carried out using a direct contact method *in vitro*. Following incubation in a suspension of GC-7 for 24 h, all treatments at different concentrations exhibited significant larvicidal potential. The collected mortality at concentrations of 50, 20, and 10% GC-7 was 95.82, 87.86, and 58.79%, respectively, which were significantly higher than that in the controls (Figure 2A). The results indicated that the cell-free culture filtrate of *P. rhodesiae* GC-7 was highly antagonistic to *M. graminicola*.

Moreover, the effect of GC-7 on *M. graminicola* egg hatching is shown in Figure 2B. The GC-7 suspension significantly reduced nematode egg hatching compared with CK at each stage of incubation. After incubation for 48 h, percentage egg hatching in the GC-7-treated and control sample was 15.61 and 29.73%, respectively, and the hatch inhibition rate was up to 47.48% in GC-7 compared to the control treatment (Figure 2B). The hatch inhibition rate of isolate GC-7 was 60.65% at 96 h after treatment. These results indicated that isolate GC-7 could significantly inhibit the hatching of *M. graminicola* eggs.

### Effect of isolate GC-7 on infection by *Meloidogyne graminicola* J2s

**Figure 3:**
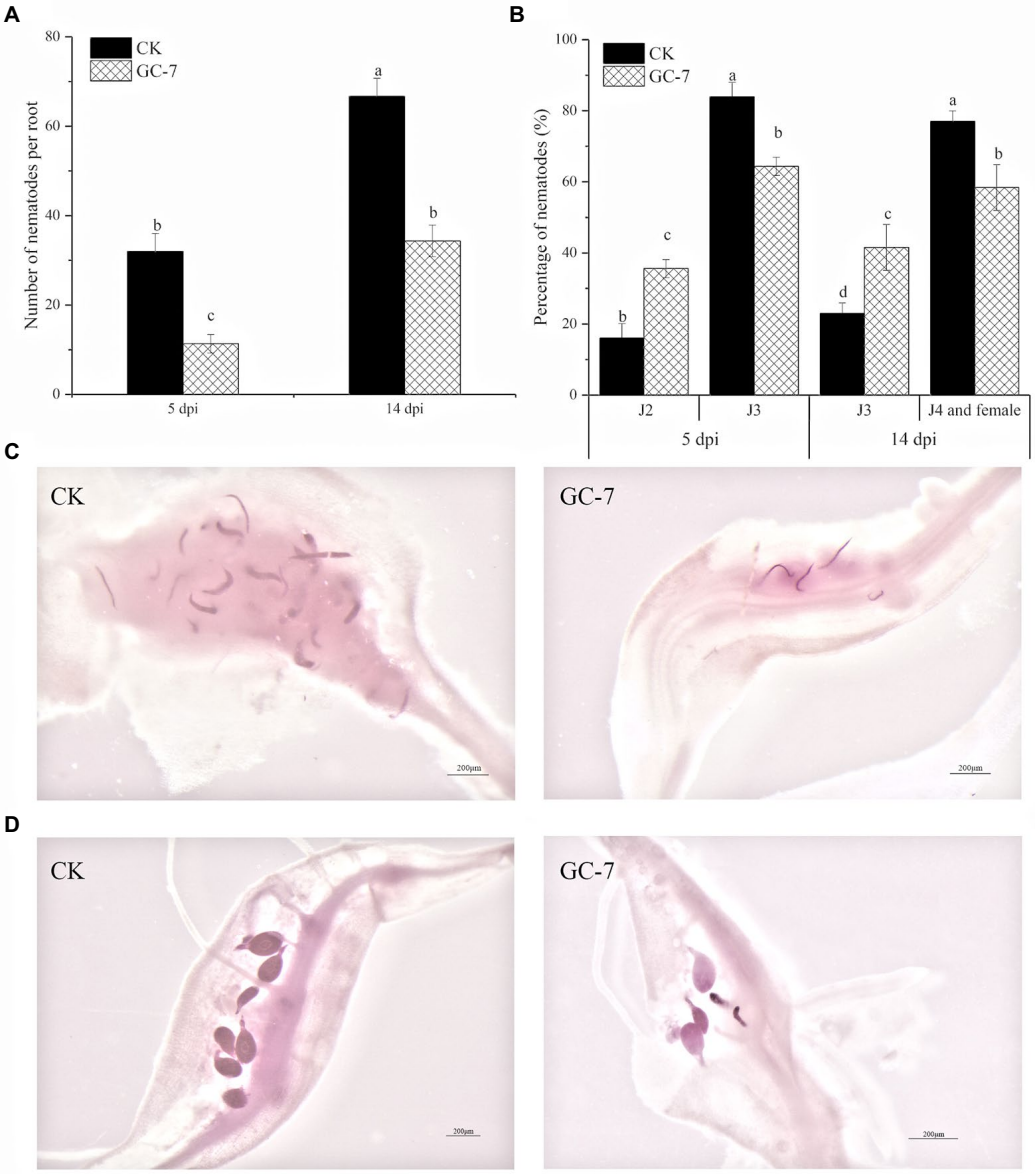
Infection and development of *M. graminicola* inside rice roots at 5 dpi and 14 dpi. **(A)** Penetration of *M. graminicola* in GC-7- and NB medium-treated (CK) rice roots. **(B)** The ratio of nematodes in rice roots at different developmental stages at 5 dpi and 14 dpi. **(C)** Nematodes in CK and GC-7-treated plants were photographed at 5 dpi. **(D)** Nematodes in CK and GC-7-treated plants were photographed at 14 dpi. The roots of rice seedlings were immersed in a 50% GC-7 strain fermentation culture (1 × 10^9^ cfu/ml) for 8 h, and roots soaked in 50% NB medium for 8 h were used as controls. Each plant was inoculated with about 100 J2s. Scale bar = 200 μm. The bars in the different graphs represent the mean ± SE of data from three independent biological replicates, each containing eight individual plants. Different letters indicate statistically significant differences (Tukey’s multiple range test at *p* ≤ 0.05).

The nematode penetration recorded at 5 and 14 days post inoculation (dpi) is shown in Figure 3. Nematode observation in roots after staining showed that the total number of nematodes was significantly lower in GC-7-treated plants compared to the untreated controls. The reduction in the number of nematodes inside GC-7-treated roots was 64.58 and 48.50% at 5 dpi and 14 dpi, respectively (Figure 3A).

The development of nematodes in GC-7-treated plants was slightly delayed compared with that in untreated control plants. At 5 dpi, the percentage of third-stage juvenile (J3) in GC-7-treated plants (64.36%) was significantly lower than that in untreated plants (83.93%). At 14 dpi, the ratio of fourth-stage juvenile (J4) and adult females was higher in untreated plants (77.01%) than in GC-7-treated roots (58.42%). By contrast, the percentage of J3s was lower in untreated plants (22.99%) than in GC-7-treated plants (41.58%) (Figures 3B–D). These data showed that isolate GC-7 not only lowered infection by *M. graminicola* but also slightly inhibited nematode development in rice roots.

### Biocontrol of *Meloidogyne graminicola* in pot-based experiments

**Figure 4:**
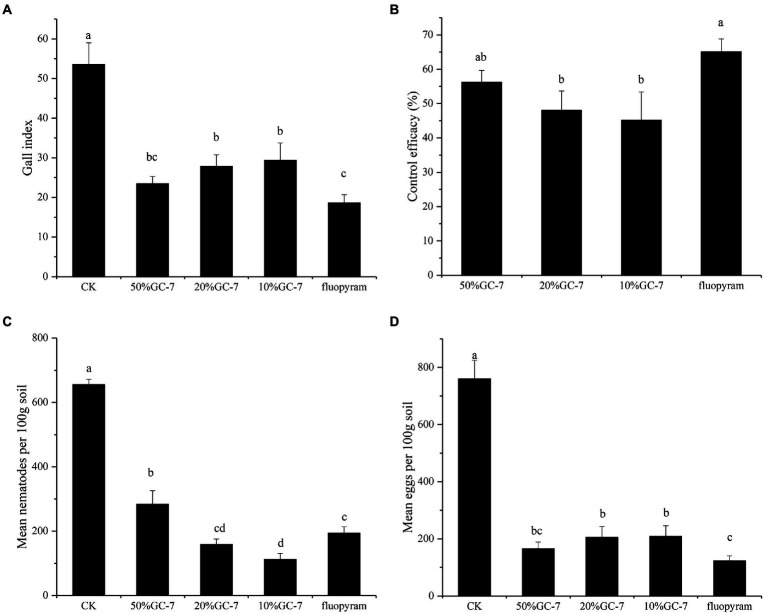
Biocontrol of *M. graminicola* in the pot-based experiment. **(A)** Gall index of GC-7 against *M. graminicola* in the pot-based experiment. **(B)** The control efficacy in different treatments. **(C)** Effects of the application of GC-7 on the final nematodes in soil. **(D)** Suppression of *M. graminicola* eggs by GC-7 in soil of pot-based experiment under greenhouse conditions. Each pot contains five two-leaf stage rice seedings, fourty millilitre of the different concentrations of the bacterial fermentation broth was applied as irrigation to each pot. The control were treated with sterilized water or fluopyram. Two days after inoculation with the bacterial fermentation broth, 2 ml of *M. graminicola* suspension (750 vigorous J2s/mL) was drenched into each pot. Vertical bars represent standard deviation of the mean values. Tukey’s multiple range test was employed to test for significant differences between treatments at *p* < 0.05. Different lowercase letters indicate significant difference between treatments (*p* < 0.05).

Pot-based experiments were carried out to further evaluate the biocontrol efficiency of isolate GC-7 *in vivo* using a soil drenching method. Here, *M. graminicola* infection was severe, forming numerous large root galls on the roots of control rice plants. By contrast, fewer and smaller galls were observed on rice roots following treatment with GC-7 fermentation broth. The average root gall index of the control group was 53.56, and the average root gall index with all dilutions (50, 20, 10%) of isolate GC-7 were 23.44, 27.81, and 29.36, respectively (Figure 4A). All concentrations of the GC-7 fermentation broth showed a high control effect on *M. graminicola* with a control efficiency of 45.19 to 56.23% (Figure 4B), However, application of different concentrations of GC-7 did not show dose effect on gall index and control efficacy. In addition, the final nematode density in soil treated with GC-7 was also significantly lower than that of the CK. Inhibition rates for treatment with 20 and 10% GC-7 fermentation broth reaching 75.84 and 82.84%, respectively, higher than treatment with fluopyram (70.42%; Figure 4C). Moreover, the number of eggs in soil treated with isolate GC-7 was lower than in the control soil, with inhibition rates reaching 72.52 to 78.18% (Figure 4D).

In addition, the effect of different dilutions of GC-7 fermentation broth on the growth of rice parasitized by *M. graminicola* showed a significant improvement in all plant growth parameters compared to the control (Table 2). Rice treated with 50% GC-7 showed the highest average total length (53.83 cm), root length (15.22 cm), and total fresh weight (1.70 g), corresponding to increases of 17.89, 22.41, and 68.98%, respectively, above control treatments. The control samples exhibited the lowest plant growth.

**Table 2:** Effects of biocontrol agents (isolate GC-7) on the growth parameters of rice plants infected with *Meloidogyne graminicola* in pot-based experiments.

Treatments	Total length (cm)	Root length (cm)	Total fresh weight (g)
GC-7 (50%)	53.83 ± 2.65a	15.22 ± 0.60a	1.70 ± 0.16ab
GC-7 (20%)	52.63 ± 2.47a	14.74 ± 0.58a	1.59 ± 0.10bc
GC-7 (10%)	49.86 ± 1.60ab	14.21 ± 0.37a	1.44 ± 0.08c
CK	45.67 ± 1.55b	12.43 ± 0.90b	1.01 ± 0.19d
Fluopyram	53.27 ± 2.74a	15.39 ± 0.49a	1.89 ± 0.05a

The data are mean ± SE from four replicates per treatment. Means followed by the same letter in the same column are not considered statistically different following Tukey’s multiple range test (*p* < 0.05).

### Efficacy in controlling nematodes in the field experiment

**Figure 5:**
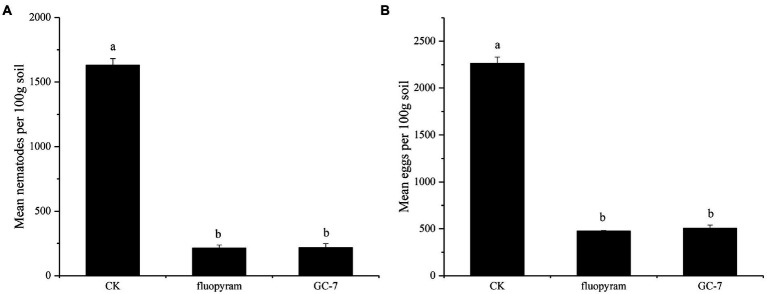
Biocontrol of *M. graminicola* in the field. **(A)** GC-7 isolate decreased the nematodes in soil. **(B)** Suppression of *M. graminicola* eggs by GC-7 in soil under field conditions. At the time of harvest (4 months after planting), the nematodes and eggs in each 100 g soil sample were isolated and counted. Vertical bars represent standard deviation of the mean values. Tukey’s multiple range test was employed to test for significant differences between treatments at *p* < 0.05. Different lowercase letters indicate significant difference between treatments (*p* < 0.05).

The field experiment was carried out using the GC-7 fermentation broth. The average root gall index corresponding to the GC-7 treatment against *M. graminicola* was 18.63 (Table 3), which was significantly lower than that of the control (45.27). GC-7 culture showed a high control effect on *M. graminicola* with a control efficiency of 58.85%. In addition, the inoculation of isolate GC-7 in *M. graminicola* infested rice fields significantly suppressed the nematode and egg populations under natural conditions (Figure 5). The final nematode population in 100 g of GC-7-treated soil and chemical nematicide fluopyram-treated soil was much lower than CK soil (Figure 5A). The number of eggs in GC-7-treated soil reduced by 77.67% compared to CK soil (Figure 5B). Concurrently, the application of GC-7 displayed a better effect on the promotion of root weight and stem base width than CK and fluopyram. At harvest, the rice yield increased by 31.98% in GC-7 treated plants compared with control (Table 3).

**Table 3:** Effects of biocontrol agents (isolate GC-7) on the growth parameters and protection of rice plants infected with *M. graminicola* under natural field conditions in 2021.

Treatments	Shoot length (cm)	Root length (cm)	Shoot fresh weight (g)	Root fresh weight (g)	Stem base width (cm)	Chlorophyll (SPAD)	Gall index	Control effect (%)	Yield(kg/ha)
CK	34.06 ± 0.94a	12.30 ± 0.10c	3.38 ± 0.20b	2.15 ± 0.17c	0.47 ± 0.03c	38.56 ± 0.59b	45.27 ± 6.41a	–	4,384 ± 863b
Fluopyram	41.10 ± 2.05a	15.20 ± 0.27a	8.85 ± 0.66a	3.69 ± 0.09b	0.53 ± 0.03b	41.03 ± 0.45a	12.34 ± 3.49b	72.73 ± 7.71a	6,084 ± 606a
GC-7	39.33 ± 1.48b	13.40 ± 0.44b	8.72 ± 0.40a	4.07 ± 0.19a	0.61 ± 0.05a	40.83 ± 0.66a	18.63 ± 3.25b	58.85 ± 7.20b	5,787 ± 462a

Data are mean ± SE for four replicates per treatment. Means followed by the same letter in the same column are not considered statistically different following Tukey’s multiple range test (*p* < 0.05).

### Enhancement of resistance-related enzyme activities

**Figure 6:**
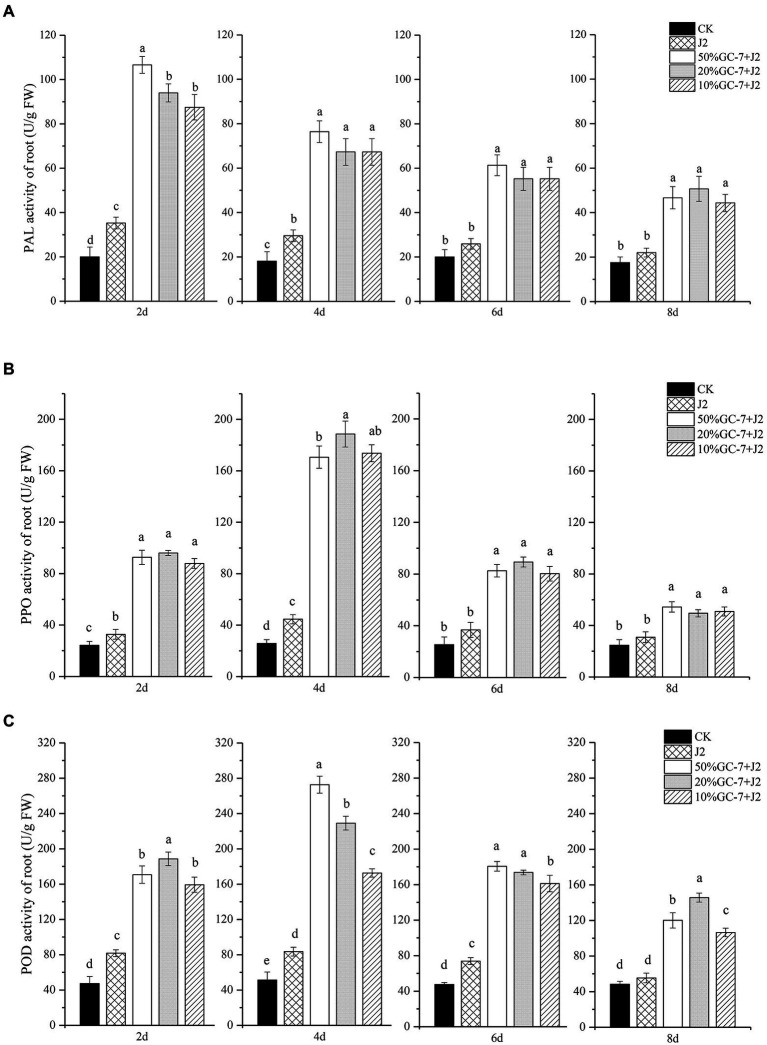
Changes of defense enzyme (PAL, PPO, POD) activities in roots of rice plants subjected to different treatments and *M. graminicola*. **(A)** PAL activity (U/g FW). **(B)** POD activity (U/g FW). **(C)** PPO activity (U/g FW) in rice roots inoculated with GC-7 fermentation broth at different dilutions and *M. graminicola.* Each pot contains five two-leaf stage rice seedings, fourty millilitre of the different concentrations of the bacterial fermentation broth was applied as irrigation to each pot. The control rice seedlings were treated with sterilized water. Each individual pot was inoculated with approximately 1,500 freshly hatched *M. graminicola* J2s 2 days later. Values are means of three replicates and vertical bars represent the standard error. All treatments are significantly different from each other at *p* < 0.05.

The quantitative changes in the defense-related enzyme activities of POD, PPO, and PAL in rice roots were investigated. Supplementation of GC-7 resulted in significant enhancement of the PAL activities in nematode-infected plants compared to CK plants and challenged with the nematode-treated (J2) plants at all times. The highest PAL activity was recorded in plants treated with 50% GC-7 + J2 at 2 dpi, which was elevated by 4.33-fold and 2.02-fold compared to CK and nematode-treated plants, respectively (Figure 6A). Rice plants inoculated with nematodes only had slightly higher PAL activity compared to the untreated control. PPO activity from 2 dpi was induced by the GC-7 fermentation broth and plants treated with almost all concentrations of bacteria showed the highest levels of PPO activity at 4 dpi. The maximum level of PPO activity was observed in plants treated with a 20% dilution of GC-7 (20% GC + J2), increasing 6.34-fold and 3.23-fold compared to CK and nematode-treated plants, respectively (Figure 6B). Untreated control plants showed the lowest enzyme activity among all treatments. A similar pattern of increased POD activity was found in bacterized plants treated with the challenge inoculation. Plants treated with each concentration of GC-7 + J2 expressed higher POD activity compared to untreated control and nematode-infected (J2) plants (Figure 6C). The enzyme activities peaked at approximately 4 dpi, then slowly decreased. The highest activity was observed in plants treated with 50% GC-7 + J2 at 4 dpi, which increased by 4.31-fold and 2.27-fold compared to CK and nematode-treated plants, respectively.

### Transcriptional levels of defense-related genes

**Figure 7:**
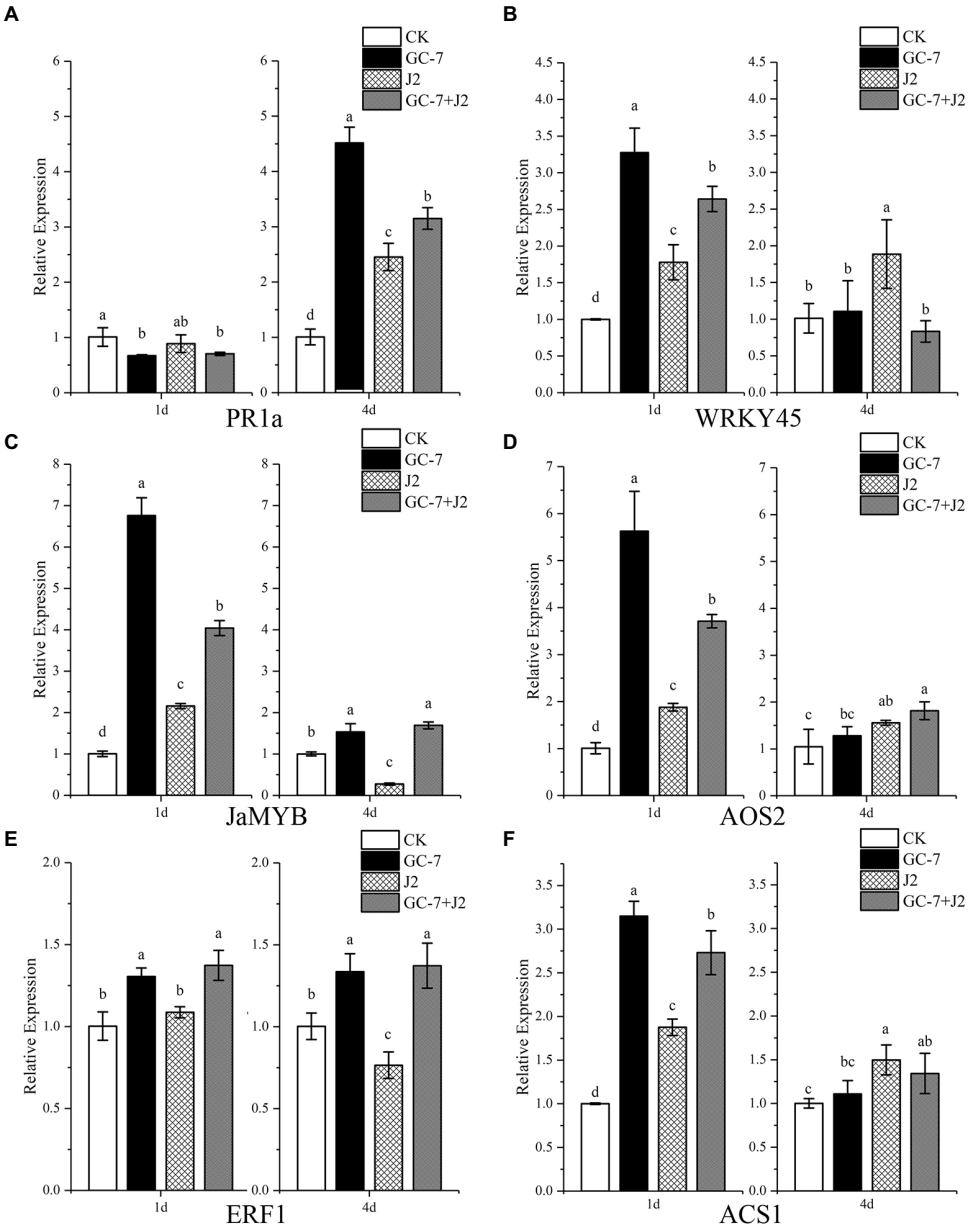
Quantitative RT-PCR analysis of genes involved in the salicylic acid, jasmonic acid, and ethylene pathways in rice roots. **(A)** Relative expression of *PR1a*. **(B)** Relative expression of *WRKY45*. **(C)** Relative expression of *JaMYB*. **(D)** Relative expression of *AOS2*. **(E)** Relative expression of *ERF1*. **(F)** Relative expression of *ACS1.* Gene expression levels were analyzed from four treatments: CK, GC-7, J2, GC-7 + J2 at 1 and 4 days after *M. graminicola* infection. Each pot contains five two-leaf stage rice seedings and was drenched with 40 ml of 50% GC-7 (1 × 10^9^ cfu/ml) or sterilized water. The individual pot was inoculated with approximately 1,500 freshly hatched *M. graminicola* J2s 2 days later. Rice seedlings were treated with sterile water as the non-treated control Error bars represent standard error of the mean values of three independent replicates from four biological replicates. All treatments are significantly different from each other at *p* < 0.05. For each gene and time point, bars with different letters indicate statistically significant differences between treatments.

We studied the relative expression levels of defense linked genes *PR1a* and *WRKY45* associated with the SA pathway, ET responsive transacting factors *ERF1* and *ACS1*, as well as JA-dependent gene markers *JaMYB* and *AOS2*, to obtain an insight into the GC-7-induced rice defense response against *M. graminicola*. The relative expression level of *PR1a* at 1 dpi decreased by 33.48% in GC-7-treated plants, 30.28% in GC-7 + J2 plants, and 12.09% in J2 plants, compared with that in CK plants. However, at 4 dpi, a significant upregulation of *PR1a* was observed for all treatments. The highest relative expression level (3.48-fold higher than in the control) was reported in plants treated with GC-7, followed by GC-7 + J2 and J2, with expression levels that were 2.12- and 1.43-fold higher, respectively (Figure 7A). A significant upregulation of *WRKY45* was observed in all treated plants at 1 dpi compared to the control. However, no differences were observed in plants treated with GC-7 and GC-7 + J2 compared with CK plants at 4 dpi (Figure 7B).

At 1 dpi, significant increases in the relative expression levels of the *JaMYB* and *AOS2* were observed in plants treated with GC-7 and GC-7 + J2 compared with control and J2-treated plants. The expression level of *JaMYB* was most notably higher in GC-7-treated plants (5.75-fold), followed by GC-7 + J2- (3.03-fold) and J2- (1.15-fold) treated plants, than in control plants (Figure 7C). In a similar pattern, *AOS2* expression level was upregulated in plants from all treatments compared to the control at 1 dpi. The highest transcriptional level was observed with GC-7 (4.59-fold), while the levels for the GC-7 + J2 and J2 treatments were 2.69- and 0.89-fold higher, respectively. At 4 dpi, the JA gene expression level in plants from the GC-7 and GC-7 + J2 treatments was slightly higher than in the CK plants (Figure 7D).

A significantly and consistently enhanced transcription of *ERF1* was induced by GC-7 in GC-7 + J2 at 1 dpi and 4 dpi, compared with that induced by the CK and J2 treatments. By contrast, the transcript level in J2-treated plants was repressed at 4 dpi compared to CK plants (Figure 7E). Transcription of *ACS1* in plants treated with GC-7, GC-7 + J2, and J2 was 2.14-fold, 1.73-fold, and 0.87-fold higher, respectively, than the control at 1 dpi (Figure 7F).

## Discussion

RKNs are a severe threat to world agriculture. Many nematicidal biocontrol agents have been discovered from microorganisms, and the application of bacteria, especially PGPR, have received increasing attention (Hu et al., 2017; Liang et al., 2019). However, the microorganisms used for controlling the rice RKN *M. graminicola* are limited. In the present study, the novel *P. rhodesiae* isolate GC-7 was isolated from the rhizosphere soil of rice plants. This isolate could efficiently kill *M. graminicola* J2s directly, as well as suppress egg hatching *in vitro*. Microorganisms can inhibit growth of PPNs through diverse processes. Production of various antagonistic compounds with inhibiting effects against PPNs is a direct mode of action. Many bacteria produce nematicidal bioactive hydrolytic enzymes, antibiotics and toxins that directly kill nematodes or inhibit proliferation (Yang et al., 2013; Mnif and Ghribi, 2015; Migunova and Sasanelli, 2021). In addition, some bacterial strains also produce nematicidal volatile organic compounds (VOC) against *Meloidogyne* (Zhao et al., 2018). The underlying mechanisms by which GC-7 restrains *M. graminicola* can be complicated. Further research is needed to determine primary nematicidal compounds of *P. rhodesiae* GC-7. Greenhouse and field tests demonstrated that GC-7 effectively controlled *M. graminicola* and promoted rice plant growth. The biocontrol effects of GC-7 might be because the isolate adheres to the rhizospheric zone and increases plant biomass to secrete stronger substances that kill nematode J2s and inhibit egg hatching (Khanna et al., 2019; Zhao et al., 2021). In addition, our data demonstrated a significant reduction in *M. graminicola* penetration in rice plants and their population densities in GC-7-treated soil. Moreover, nematode development was inhibited in GC-7-treated plants compared with that in non-treated plants. These results are consistent with previous reports of PGPR and other beneficial microbes (Liu et al., 2018, 2019; Zhou et al., 2021). The number of egg masses was considered as the infective ability of the nematode as it shows the number of J2s that were able to penetrate and infect the root tissue and develop into egg-laying females (Ghareeb et al., 2020). Here, the egg populations in soil treated with GC-7 under greenhouse and natural conditions were significantly decreased compared with control. Therefore, our study aligns with the three key principles of biological control proposed by Stenberg et al. (2021), and indicates that *P. rhodesiae* isolate GC-7 could be used as a potential biological nematicide against *M. graminicola*.

Several rhizobacteria can enhance plant resistance against pathogens by activating defense-related enzymes (Chen et al., 2009; Fatma et al., 2014; Narendra Babu et al., 2015; Salla et al., 2016; Rais et al., 2017). PAL has been reported to be involved in plant defense mechanisms as it is the first enzyme in phenylpropanoid metabolism and related to the biosynthesis of phenolics and SA (Jain and Choudhary, 2014; Di Lelio et al., 2021). PPO plays an important role in phenol metabolism related to the synthesis of lignin and quinone compounds, which can inhibit the invasion of pests and diseases (Ju et al., 2013). POD activity is important in lignin accumulation and ROS production, both important components of a plant’s active defense response against pathogens (de Oliveira et al., 2021). Our data indicated that, following *M. graminicola* infection, POD, PPO, and PAL activities showed more rapid and greater increases in the rice roots of GC-7-treated plants than in the controls, further revealing the mechanism underlying nematode control by GC-7. The highest PAL activity in GC-7 fermentation broth-treated plants were observed at 2 dpi, whereas POD and PPO activities peaked at 4 dpi. The results suggest that isolate GC-7 can significantly enhance such defense enzymes and probably other defense compounds, leading to systemic resistance in plants. These findings are consistent with those of previous studies, which showed increasing levels of defense enzymes during the early stages of defense, thus playing a crucial role in plant host resistance (Jain and Choudhary, 2014; Kurth et al., 2014). In addition, PAL, PPO, and POD activities in non-bacterized plants increased slightly after the nematode challenge, indicating that some increased enzyme activity is a natural response of infected plants susceptible to nematode attack. This finding is similar to those of previous studies, in which plants subjected to pathogen control showed higher enzyme activities of PAL, POX, and PPO than the untreated control (Jetiyanon, 2007; Chen et al., 2009). However, the level of the increase was too low to overcome nematode invasion.

The major defense mechanisms of plants are regulated through SA, JA, or ET signaling pathways (Shoresh et al., 2005; Rasool et al., 2021). Certain related defense genes are activated by various factors, consequently inducing systemic resistance against disease. ISR often relies on pathways regulated by JA/ET. *JaMYB* and *AOS2* are marker genes for JA-dependent signaling pathways, whereas *ERF1* and *ACS1* are markers of the ET signaling pathway (Leonetti et al., 2017; Salman et al., 2022). Previous studies on rice have also shown that the exogenous application of plant hormones and antioxidants induces systemic defense against *M. graminicola,* and that the JA pathway plays a pivotal role in the induction of defense against RKN (Nahar et al., 2011; Singh et al., 2020; Chavan et al., 2022). Conversely, abscisic acid (ABA), brassinosteroids (BRs), gibberellic acid, and strigolactones promote the susceptibility of rice to *M. graminicola* infection by interacting antagonistically with the JA pathway (Nahar et al., 2012; Yimer et al., 2018; Lahari et al., 2019). Rhizobacteria may act against nematodes through ISR in plants (Ryan et al., 2015; Migunova and Sasanelli, 2021); however, little is known about the role of bacteria in the interaction between rice plants and *M. graminicola.* We studied the influence of isolate GC-7 on defense-associated gene expression in rice plants either challenged with *M. graminicola* or free from infection. The qRT-PCR results indicated that rice seedlings treated with isolate GC-7 only greatly enhanced the subsequent expression of *JaMYB*, *AOS2, ERF1*, and *ACS1* (especially JA signaling pathway genes *JaMYB* and *AOS2*) at 1 dpi. The JA pathway, in particular, may play an important role in inducing defense against *M. graminicola*. Moreover, nematode infection provoked a marked increase of these gene transcript levels in the roots of plants pre-treated with isolate GC-7 compared with uninoculated (J2-treated) plants at 1 dpi. *JaMYB* and *ERF1* transcript levels remain high, whereas *AOS2* transcript levels show a slight increase at 4 dpi. The high expression of defense-related genes in GC-7 treated roots suggests that rice seedlings respond rapidly to *M. graminicola* infection, indicating GC-7 ISR and the JA- and ET- dependent ISR pathways are involved in root resistance to nematode infection by isolate GC-7 at an early stage.

*PR1a* and *WRKY45* are markers of the SA signaling pathway and are major characteristic proteins of SAR (Salman et al., 2022). *WRKY45* and *PR1a* expression were increased in GC-7- and J2 + GC-7-treated plants compared to control and J2-treated plants at 1 dpi and 4 dpi, respectively, indicating that the defense response of rice to *M. graminicola* mediated by the SA signaling pathway was also activated. Our result is similar to a previous finding, where certain PGPRs, including *Bacillus* spp., *Pseudomonas* spp., and fungi such as *Trichoderma*, can activate SAR and facilitate the expression of characteristic *SAR* genes in plants, thereby enhancing plant systemic resistance (Subedi et al., 2020; Rasool et al., 2021; TariqJaveed et al., 2021). Previous reports have shown that *Trichoderma* could reduce *Meloidogyne* by triggering host defense, which was attributed first to SA-regulated defense that limited the root invasion of nematodes, then to enhanced JA-regulated defense (Martinez-Medina et al., 2017; Medeiros et al., 2017). Similarly, it has been reported that *Microbacterium maritypicum* Sneb159 induced an interaction between the SA-dependent SAR pathway and JA-dependent ISR pathway to defend against *H. glycines* (Zhao et al., 2019). In addition, treatment with isolate GC-7 suppressed the expression of *PR1a* at 1 dpi compared to CK plants, which may be to allow the colonization of rice roots at the early stage. Our finding was similar to SA-mediated plant defense, which is generally inhibited by certain microorganisms to achieve a compatible interaction and host colonization (Leonetti et al., 2017; Zhao et al., 2019). The results implied that GC-7 might also activate the SA signaling pathways to induce plant defense against *M. graminicola*. Our report is the first to suggest that *P. rhodesiae* as a PGPR inducing protection against *M. graminicola* of rice plants involves both ISR and SAR mechanisms.

In conclusion, we isolated the novel *P. rhodesiae* isolate GC-7 from the rhizoplane of rice plants. Isolate GC-7 exhibited direct nematicidal and egg inhibition against *M. graminicola*, as well as effectively prevented nematode invasion and delayed the development of *M. graminicola* in rice roots. Pot- and field-based tests showed that isolate GC-7 significantly reduced the gall index and presented remarkable growth-promoting properties in rice plants. In addition, GC-7 also activated resistance-related gene expression and defense enzyme activity to enhance plant resistance against *M. graminicola*. Our results suggest that isolate GC-7 employs multiple anti-nematode mechanisms and could be a potential biocontrol agent for *M. graminicola*.

## Data availability statement

All datasets generated for this study are included in the article/Supplementary material, further inquiries can be directed to the corresponding author.

## Author contributions

ZD and SY: conception of the work. SY, RY, XL, YL, and ZY: collection of data. SY, RY, ZD, and YM: analysis of data. SY, ZD, and YM: writing of manuscript. All authors have read and agreed to the published version of the manuscript.

## Funding

This work was supported by the National Natural Science Foundation of China (grant nos. 32001879 and 31872038) and Natural Science Foundation of Hunan Province (grant no. 2022JJ30302).

## Conflict of interest

The authors declare that the research was conducted in the absence of any commercial or financial relationships that could be construed as a potential conflict of interest.

## Publisher’s note

All claims expressed in this article are solely those of the authors and do not necessarily represent those of their affiliated organizations, or those of the publisher, the editors and the reviewers. Any product that may be evaluated in this article, or claim that may be made by its manufacturer, is not guaranteed or endorsed by the publisher.

## Footnotes

^1^ http://www.ncbi.nlm.nih.gov/
