# Supplementary material for: Biocontrol potential of Pseudomonas rhodesiae GC-7 against the root-knot nematode Meloidogyne graminicola through both antagonistic effects and induced plant resistance
Source: Front Microbiol. 2022 Oct 13;13:1025727. doi: 10.3389/fmicb.2022.1025727 (PMC9651087; doi:10.3389/fmicb.2022.1025727)
Supplement: Supplementary file 1 [file Table_1.DOCX]

Supplementary Material

# Supplementary Table

**Supplementary Table S1.** **List of primers for qRT-PCR**

| Primer | GenBank accession/  Locus number | Primer sequences(5′ to 3′) |
| --- | --- | --- |
|  |  |  |
| *Actin* | AK060893 | F: GATCACTGCCTTGGCTCCTA |
|  |  | R: CCGGTAGAGCGGATACGAC |
| *PR1a* | AP003877.3 | F: TCGTATGCTATGCTACGTGTTT |
|  |  | R: CACTAAGCAAATACGGCTGACA |
| *WRKY45* | Os05g0322900 | F: AATTCGGTGGTCGTCAAGAA |
|  |  | R: AAGTAGGCCTTTGGGTGCTT |
| *AOS2* | Os03g12500 | F: CAATACGTGTACTGGTCGAATGG |
|  |  | R: AAGGTGTCGTACCGGAGGAA |
| *JaMYB* | AY026332 | F: GAGGACCAGAGTGCAAAAGC |
|  |  | R: CATGGCATCCTTGAACCTCT |
| *ERF1* | LOC4336571 | F: AAGGGTCATAATTCGCGTCA |
|  |  | R: TCCACACCACAAGACATCGT |
| *ACS1* | LOC_Os03g51740 | F: GATGGTCTCGGATGATCACA |
|  |  | R: GTCGGGGGAAAACTGAAAAT |
